# 

**Published:** 1880-07

**Authors:** 


### CONTENTS:

ART. I.—Transactions of the Odontological Society of Great Britain..........97
ART. II.—Operative Dentistry. By Isaac J. Wetherbee, D. D. S..........125
ART. III.—Call for a Convention to Organize a National Dental Association..........131
ART. IV.—Bromide of Ethyl..........133
ART. V.—The National Dental Association..........134

### EDITORIAL, ETC.

Death of Dr. Robert Arthur..........136

### OBITUARY.

Thomas Bell, F. R. S., &c..........140
Robert Arthur, M. D., D. D. S..........140

### MONTHLY SUMMARY.

A Nerve Stimulant..........141
Use of Chloroform in Diseases of the Heart..........142
The Setting of Plaster..........143
A Form of Land Scurvy..........143
A New Narcotic..........144
Apparent Death as a Result of Asphyxia..........144

